# The Open-Book Technique With Sliced Biceps Tendon Autograft Augmentation in the Reconstruction of the Subscapularis in Shoulder Arthroplasty

**DOI:** 10.1016/j.eats.2024.103345

**Published:** 2024-12-03

**Authors:** Amadeo Touet, Katja Rüttershoff, David Endell, Markus Scheibel

**Affiliations:** a Department of Shoulder and Elbow Surgery, Schulthess Clinic Zurich, Zurich, Switzerland; b Department of Orthopedics and Trauma Surgery, University Hospital Bonn, Bonn, Germany; c University Hospital Basel, Basel, Switzerland; d Center for Musculoskeletal Surgery, Charité-University of Berlin, Berlin, Germany

## Abstract

Postoperative insufficiency of the subscapularis can diminish clinical outcome after shoulder arthroplasty. Poor tendon tissue quality and prior surgery can impair the possibility of reconstruction and healing, as well as the postoperative range of motion and strength. Patch augmentation has been investigated for soft tissue augmentation in the glenohumeral joint utilizing different graft materials. Recently, the surgical technique using compressed biceps tendon autograft was introduced for the subscapularis augmentation following shoulder arthroplasty. However, the viable cell potential after mechanical compression remains uncertain. Therefore, we propose an augmentation technique of the subscapularis tendon using a sliced autograft of the long head of the biceps tendon. This technique can offer a simple, replicable, and cost-effective autologous tendon patch augmentation after subscapularis tenotomy in the setting of total shoulder arthroplasty.

Insufficient healing of the subscapularis tendon after total shoulder arthroplasty can cause persistent shoulder pain and impairment.^1^ Postoperative repair failure after subscapularis takedown is high, with reported retear rates of 23% to 60%.^1^, ^2^, ^3^ Patients with subscapularis insufficiency experience reduced range of motion and strength for internal rotation, regardless of the type of shoulder arthroplasty.^3^^,^^4^ Various techniques aim to preserve the continuity of the subscapularis tendon. However, this limits the surgical approach and intraoperative joint exposure.^5^^,^^6^ Hence, a deltopectoral approach for total shoulder arthroplasty is commonly performed, followed by a subscapularis takedown using peel-off, lesser tuberosity osteotomy, or tenotomy.^3^^,^^7^ Anatomic total shoulder arthroplasty in particular relies on an intact and balanced anteroposterior muscular force-couple for joint centering and stabilization. Nonetheless, subscapularis tendon integrity is also advantageous in reverse total shoulder arthroplasty in terms of strength, range of motion, and clinical outcomes.^8^

Recently, Denard et al.^9^ presented a biological augmentation technique of the subscapularis tendon, using a compressed proximal long head of the biceps (LHB) tendon. A similar technique has been introduced for augmentation in arthroscopic rotator cuff repair.^10^^,^^11^ However, there are no valid long-term data on the viability of the tenocytes and correspondingly on the biological activity after mechanical compression with these high pressures. In this case report, we present an augmentation technique using a sliced autograft of the proximal LHB for patients at risk of tendon insufficiency after subscapularis tenotomy in total shoulder arthroplasty.

## Surgical Technique

### Indication

The biceps slice augmentation technique is a valuable option, regardless of whether the surgical approach involves subscapularis peel-off or tendon takedown, since it provides additional biological augmentation to enhance tendon healing. This technique may be indicated for patients at risk for postoperative subscapularis insufficiency, particularly with pre-existing weakness in clinical examination, or radiographic and intraoperative signs of tendon degeneration. An intact portion of at least 2 cm of the LHB tendon is recommended to ensure feasibility of the procedure. Moderate synovitis is not an exclusion criterion for biological augmentation, as long as the base of the graft remains intact.^12^

### Operative Setup and Subscapularis Augmentation

In a beach-chair position, a standard deltopectoral approach for joint exposure is performed. The subscapularis and the anterior capsule are detached approximately 5 mm medial to the insertion at the lesser tuberosity, leaving a tendon stump laterally. At least 4 No. 2 nonabsorbable sutures (i.e., FiberWire; Arthrex) are used to secure the subscapularis tendon in a modified Mason-Allen technique used for later reattachment. Implantation of the total shoulder arthroplasty is performed according to preoperative planning. By default, the LHB tendon is routinely tenotomized at the insertion at the superior labrum, followed by retraction into the bicipital groove. It is now possible to harvest the tendon and store it in a gauze soaked in sterile saline solution for later use. Alternatively, this step can also be performed after implantation of the endoprosthesis and reconstruction of the subscapularis in a tendon-to-tendon technique with 4 of the previously placed FiberWire sutures.

**Fig 1:**
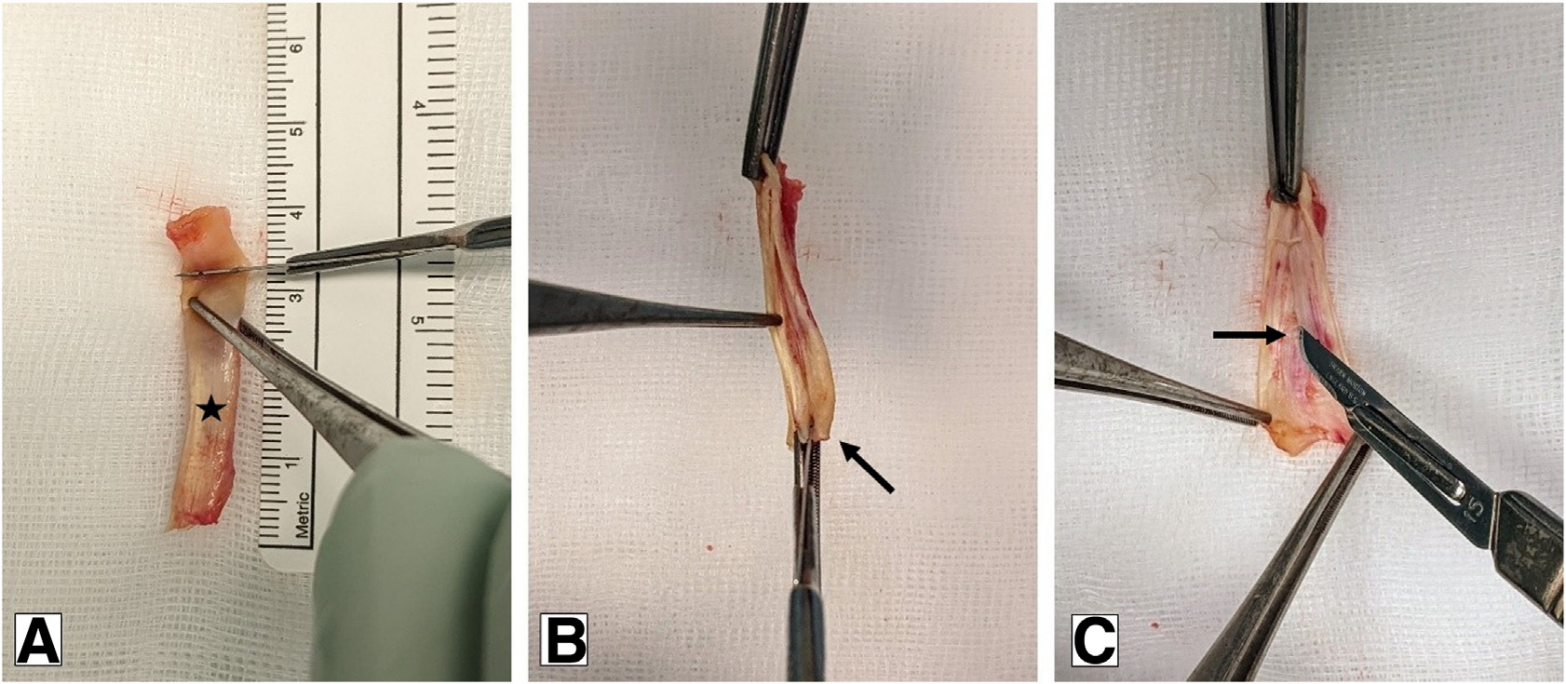
Slicing of the proximal long head of the biceps tendon. (A) Customization of the harvested long head of the biceps tendon (★). (B) Longitudinal slicing along the short side of the elliptically shaped tendon. (C) Open the book without cutting through the entire tendon.

**Fig 2:**
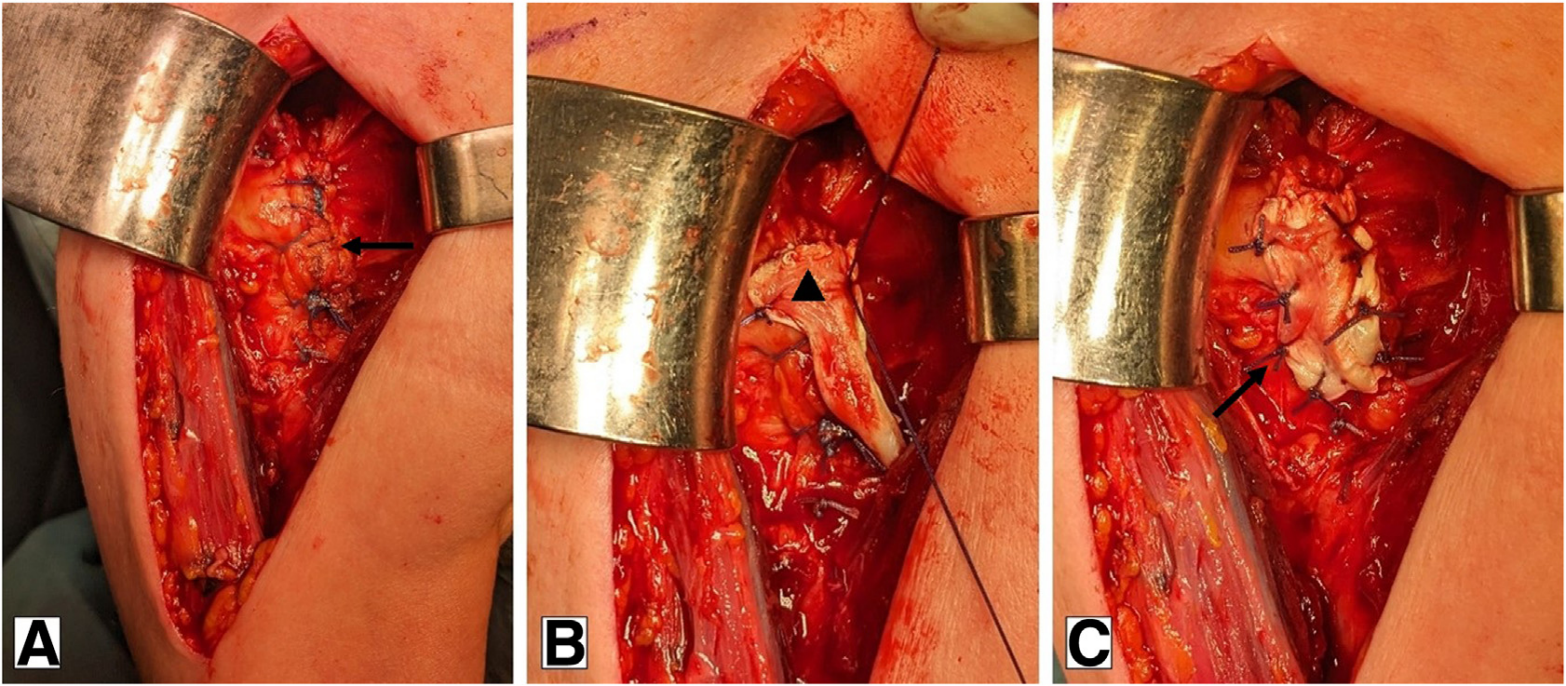
Augmentation with the sliced long head of the biceps tendon. Beach-chair position using a standard deltopectoral approach (right shoulder). (A) After implantation of the arthroplasty, a tendon-to-tendon repair of the subscapularis tendon is performed. An additional No. 1 absorbable running stitch (i.e., Vicryl; Ethicon) is used to eliminate any dog-ear deformities and create a solid end-to-end repair construct. (B, C) Intraoperative positioning of the sliced biceps autograft (▴) over the reconstructed subscapularis and fixation with single stiches.

To harvest the LHB tendon, the arm is placed in a neutral position with 90° of elbow flexion. Palpating the bicipital groove the LHB tendon is identified, liberated, and cut at the level of the insertion of the pectoralis major tendon. The distal end of the tendon is then sutured with a Krackow technique and fixed against the pectoralis major tendon. The graft preparation can be performed meanwhile by the assistant. The harvested LHB is fixed with forceps, and a longitudinal incision is performed along the short side of the elliptically shaped tendon. The incision is made close to the opposite side without cutting entirely through the whole tendon, allowing the tendon to be opened like a book (Fig 1). In a sample of 12 patients, the unfolded tendon had a mean (SD) size prior augmentation of 3.4 (0.28) × 1.5 (0.25) cm. The autograft is then stretched over the reconstructed subscapularis, with each of the 4 ends anchored with a single stitch (e.g., Vicryl No. 0) and secured with additional stitches to create a stable augmentation (Fig 2). Alternatively, a circular simple running suture can be used. In the previously mentioned sample, a mean (SD) time of 5.4 (1.21) minutes was recorded for harvesting, preparation, and augmentation of the graft. Finally, wound closure is performed in a standardized fashion.

The postoperative rehabilitation follows a standardized protocol. The sliced biceps augmentation does not require extra movement restrictions. For LHB tenodesis, passive flexion and supination of the elbow against resistance should be limited for the first 4 weeks. The arm is immobilized for 4 to 6 weeks with immediate passive mobilization. Rehabilitation is continued by a stepwise and pain-adapted increase of assisted motion exercises and followed by active motion restoration. Strength training is implemented after range of motion has been restored. The slicing and augmentation process is demonstrated in Video 1.

## Discussion

Biological subscapularis augmentation using sliced biceps autograft is a simple, efficient, and promising technique for patients at risk of subscapularis insufficiency after total shoulder arthroplasty. An initial successful tendon repair after subscapularis tenotomy was reported to have a failure rate of 60% at the 12-month follow-up.^13^ Even though clinical impact of deficiencies of subscapularis continuity has not been clearly defined yet, these high rates of failed tendon repair indicate insufficient repair techniques in deltopectoral shoulder surgical approaches.^14^

The LHB tendon is frequently used for biological augmentation.^15^^,^^16^ Vital cells are necessary for optimal healing and thus sufficient augmentation. In this context, the tendon-derived stem cells found in the LHB tendon are of particular interest as they are biologically highly active cells with a high regenerative potential.^12^ Therefore, the primary question in biological augmentation techniques revolves around the graft processing technique. We hypothesize that the slice technique minimizes destruction of the microarchitecture of the tendon with its organized parallel-oriented collagen structure, potentially preserving more viable cells that are essential for regeneration and subsequent augmentation.

There is currently a lack of long-term clinical data on the viability of tenocytes in LHB patches after mechanical compression with high forces. Using porous LHB scaffolds with a surgical graft expander, Colbath et al.^17^ discovered a significantly lower percentage of viable tenocytes compared to native LHB tendon (38.5% vs 68.9%). Another study found no significant difference in the viability of the tenocytes after compression, although it is important to note that this evaluation was only conducted at a single stage postcompression, and long-term observation is lacking.^18^

Using autologous tissue like the LHB tendon prevents autoimmune-triggered inflammatory reactions. It is cost-effective and readily available in most cases, and no additional instruments beyond those already available are required. It is not technically demanding and does not significantly affect the operating time. If necessary, graft preparation can also be performed by an assistant at the back table. Furthermore, the size of the graft can be adapted to the individual circumstances, depending on the tendon length harvested. Slicing almost doubles the surface area, allowing an appropriate augmentation of the craniocaudal extent of the upper two-thirds of the subscapularis tendon, which is in particular at risk of subscapularis insufficiency and degenerative alterations. Theoretically, application of augmentation for the lower part, the musculomembranous insertion, is possible as well but rarely indicated for overall low rates of tendon insufficiency and the typical cranial retear pattern.^14^^,^^19^ In contrast, the maximum size of a compressed biceps patch is limited depending on the compression tray used.^9^^,^^15^ In terms of mechanical aspects, augmentation with LHB autografts results in a stronger reconstruction, as shown by load-to-failure measurements after subscapularis peel repair obtained in a cadaver study.^20^

A major limitation of the open-book technique is the variability in the quality of the LHB tendon and its general availability. Severe degeneration may affect the quality of the graft or lead to insufficient length, restricting its use in some patients. Likewise, this technique is not applicable in patients who have undergone prior procedures involving the LHB tendon, such as tenotomy or tenodesis. Although the technique itself is relatively quick, it nevertheless prolongs the overall operating time. Finally, there are currently no long-term clinical data on graft outcome or viability, highlighting the need for further research. Table 1 summarises the pearls and pitfalls associated with this technique.

**Table 1:** Key Technical Insights and Overall Benefits (Pearls) and Potential Challenges and Limitations (Pitfalls) of the Sliced Biceps Tendon Autograft Technique for Subscapularis Augmentation

Pearls	Pitfalls
Biological augmentation with a simple and replicable technique Autologous tissue with no risk of immune rejection or inflammatory reactions Graft slicing preserves the microarchitecture and potential for its regeneration Biological augmentation without relevant surgical risks and low donor site morbidity The graft size can be customized Graft preparation can be done by the assistant at the back table without any relevant loss of time Cost-effective, using autologous tissue without additional implants/instruments	Limited graft size, depending on the available LBS tendon Variable LBS tendon quality—not suitable for patients with severe tendon damage/degeneration or prior LBS procedures (tenotomy/tenodesis) Risk of damaging graft during preparation—cutting through the tendon Limited long-term data on tenocyte viability

LHB, long head of the biceps.

The sliced biceps autograft combines the advances of a mechanical augmentation and the maximum biological potential of the harvested tendon with low donor site morbidities. This simple, reproducible, and cost-effective technique may overcome some limitations of existing methods by preserving the integrity and viability of the tendon. Further clinical studies are needed to investigate the effectiveness of reducing subscapularis insufficiency or retearing.

## Disclosures

The authors declare the following financial interests/personal relationships which may be considered as potential competing interests: D.E. is a consultant for Stryker. M.S. is a consultant for Arthrex and Stryker. All other authors (A.T., K.R.) declare that they have no known competing financial interests or personal relationships that could have appeared to influence the work reported in this paper.

## Supplementary Data

Video 1 This video demonstrates the open-book technique using a sliced proximal long head of the biceps (LHB) tendon autograft for subscapularis augmentation following shoulder arthroplasty. The patient is positioned in the beach-chair position, and a standard deltopectoral approach is used (left shoulder). The procedure can be divided into 3 key steps: (1) harvesting the LBS tendon, (2) slicing the LBS tendon by making a longitudinal incision along the short side of the elliptically shaped tendon without cutting completely through, and (3) augmentation, where the graft is stretched over the reconstructed subscapularis tendon and secured with single stiches. The video highlights the indication and key steps, including tendon preparation and augmentation.
